# Genomic Infectious Disease Epidemiology in Partially Sampled and Ongoing Outbreaks

**DOI:** 10.1093/molbev/msw275

**Published:** 2017-01-19

**Authors:** Xavier Didelot, Christophe Fraser, Jennifer Gardy, Caroline Colijn

**Affiliations:** 1 Department of Infectious Disease Epidemiology, Imperial College London, Norfolk Place, London, United Kingdom; 2 Oxford Big Data Institute, Li Ka Shing Centre for Health Information and Discovery, Nuffield Department of Medicine, University of Oxford, Oxford, United Kingdom; 3 Communicable Disease Prevention and Control Services, British Columbia Centre for Disease Control, Vancouver, British Columbia, Canada; 4 School of Population and Public Health, University of British Columbia, Vancouver, British Columbia, Canada; 5 Department of Mathematics, Imperial College, London, United Kingdom

**Keywords:** genomic epidemiology, transmission analysis, infectious disease outbreak

## Abstract

Genomic data are increasingly being used to understand infectious disease epidemiology. Isolates from a given outbreak are sequenced, and the patterns of shared variation are used to infer which isolates within the outbreak are most closely related to each other. Unfortunately, the phylogenetic trees typically used to represent this variation are not directly informative about who infected whom—a phylogenetic tree is not a transmission tree. However, a transmission tree can be inferred from a phylogeny while accounting for within-host genetic diversity by coloring the branches of a phylogeny according to which host those branches were in. Here we extend this approach and show that it can be applied to partially sampled and ongoing outbreaks. This requires computing the correct probability of an observed transmission tree and we herein demonstrate how to do this for a large class of epidemiological models. We also demonstrate how the branch coloring approach can incorporate a variable number of unique colors to represent unsampled intermediates in transmission chains. The resulting algorithm is a reversible jump Monte–Carlo Markov Chain, which we apply to both simulated data and real data from an outbreak of tuberculosis. By accounting for unsampled cases and an outbreak which may not have reached its end, our method is uniquely suited to use in a public health environment during real-time outbreak investigations. We implemented this transmission tree inference methodology in an R package called TransPhylo, which is freely available from https://github.com/xavierdidelot/TransPhylo.

## Introduction

Infectious disease epidemiology is increasingly incorporating genomic data into routine public health practice, using genome sequencing for diagnosis, resistance typing, surveillance, and outbreak reconstruction. In the latter use case, we can draw inferences about the order and direction of transmission based on the presence of mutations common to multiple pathogen isolates (Croucher and Didelot 2015; Gilchrist et al. 2015). While early works in this area assumed that pathogen genomes from a transmission pair should be identical or near-identical, a number of genomic outbreak investigations revealed the complicating factor of within-host evolution (Ypma et al. 2013; Romero-Severson et al. 2014; Worby et al. 2014).

Many important bacterial pathogens have periods of latency, chronic infection, or prolonged asymptomatic carriage, all of which contribute to the generation of within-host genetic diversity (Didelot et al. 2016). *Staphylococcus aureus* is a canonical example, in which single hosts can harbor multiple distinct lineages of the pathogen, each of which may be transmitted onwards (Young et al. 2012; Golubchik et al. 2013; Harris et al. 2013; Tong et al. 2015; Paterson et al. 2015; Azarian et al. 2016). In scenarios where a single host harbors substantial diversity, it can be difficult to infer which other hosts they infected—different lineages may have been transmitted at different points during the donor’s infection and the genome sequenced from the donor may only represent a single lineage captured at the time a diagnostic sample was taken and not the complete set of lineages present within that individual (Didelot et al. 2012, 2013). Indeed, simulation studies have shown that if within-host diversity is ignored, incorrect inferences can be drawn about the transmission events that occurred within an outbreak (Romero-Severson et al. 2014; Worby et al. 2014; Worby and Read 2015).

We have previously introduced a framework for inferring person-to-person transmission events from genomic data that consider within-host genetic diversity (Didelot et al. 2014). We use the genomic data to build a time-labeled phylogeny, which we divide into subtrees, each of which captures the variety of lineages that were present within each host. In other words, the phylogeny is colored with a unique color for each host, with each transmission event represented as a change in color along a branch. We originally used a simple susceptible-infected-recovered (SIR) model to evaluate the probability of the transmission tree, and we recently showed we can extend our approach to incorporate other types of epidemiological models (Hatherell et al. 2016). A similar approach, developed independently (Hall et al. 2015), couples phylogeny construction and transmission tree inference into a single step.

The main limitation of these previous methods is that they assume that all outbreak cases have been sampled and sequenced and that the outbreak has reached its end. These assumptions greatly simplify transmission tree inference, but do not reflect epidemiological reality. An outbreak is rarely completely sampled—cases may not be reported to public health or they may not have nucleic acid available for sequencing—and genomic epidemiology investigations are frequently unfolding in real-time, meaning an outbreak is being analyzed before it is ended. The few methods that can deal with unsampled cases do so at the cost of assuming no within-host diversity (Jombart et al. 2014; Mollentze et al. 2014). Here, we introduce a new Bayesian method for inferring transmission events from a timed phylogeny that can be applied to outbreaks that are partially sampled, ongoing, or both. We solve two problems that arise from these sampling issues: The complexity of calculating the probability of an observed transmission tree under these conditions, and the difficulty in exploring the posterior distribution of possible transmission trees given a phylogeny. Our method also permits the inference of when these transmission events occurred; when coupled with the person-to-person inference, this results in a comprehensive and epidemiologically actionable outbreak reconstruction. We apply our new approach to both simulated datasets and a real-world dataset from the genomic investigation of a tuberculosis outbreak in Hamburg, Germany.

## Methods

### Overview of Inference Strategy

We use a two-stage approach, first constructing a timed phylogenetic tree P on the observed sequences and then overlaying transmission events (Didelot et al. 2014). Let T be the transmission tree, P be the timed phylogenetic tree, *θ* be the parameters of the transmission and sampling model, and *N_eg_* the within-host effective population size.

(1) $$P(\theta ,N_{e}g,T|P)\propto P(P|N_{e}g,T)P(T|\theta )P(\theta )P(N_{e}g)$$

We compute $P(P|N_{e}g,T)$ by separating P into independent parts, each of which evolves in a different individual host (Didelot et al. 2014; Hall et al. 2015), see below. This separability relies on the assumption of a complete transmission bottleneck, meaning that that within-host genetic diversity is lost at transmission, as is commonly assumed in this context. The first challenge here is therefore to compute P(T|θ) for a general model of transmission: One that allows for both unsampled cases and varying levels of infectivity throughout the course of infection, which is representative of the biological reality for many pathogens. We first illustrate how to do this in a scenario where the outbreak is over; this is a convenient assumption mathematically and makes the derivation simpler. We then proceed to the case where data collection ends at a fixed time before the end of the outbreak, as is the case when analyzing an ongoing outbreak.

### Basic Epidemiological Model

The epidemiological process we consider is a stochastic branching process in which each infected individual transmits to secondary cases called offspring (Becker 1977; Farrington et al. 2003). The number of offspring for any infected individual is drawn from the offspring distribution α(k) and we follow previous studies (Lloyd-Smith et al. 2005; Grassly and Fraser 2008) in assuming that it is a negative binomial distribution with parameters (*r*, *p*). This choice allows individuals to have different rates at which they are in contact with others (Gamma-distributed) combined with a Poisson distribution of secondary infections given their individual rate. The mean of this distribution is called the reproduction number (Anderson and May 1992), which is constant and equal to R=rp/(1−p), and the probability of having *k* offspring is $\alpha (k)=\left(\begin{array}{c} k+r-1\\ k \end{array}\right)p^{k}{\left(1-p\right)}^{r}$. The time span between the primary and any secondary infection is drawn from the generation time distribution γ(τ), where *τ* is the time since infection of the primary case. The generation time distribution can take any form (Fine 2003) but a Gamma distribution is often used (Wallinga and Lipsitch 2007).

### Finished Outbreak Scenario

We first consider the situation where an outbreak follows the model above until there are no more infected individuals; we refer to this as a finished outbreak and we use the star subscript (*) to denote the mathematical quantities associated with this scenario. In this situation, all individuals are sampled with the same probability *π* and the time span from infection until sampling has distribution denoted σ(τ),which can in principle take any form, but for which we will use a Gamma distribution in practice by analogy with the generation time distribution γ(τ). We want to calculate the probability of a transmission tree $p_{*}(T)$. This requires some preliminary quantities.

We say that an infected individual is “included” if they are part of the transmission tree by being either sampled or by leading through transmission to at least one sampled descendant. Otherwise, we say that an infected individual is “excluded”. Let $\omega_{*}$ be the probability of being excluded. This means the individual and all of their descendants are unsampled. Considering the number of offspring *k*, we have that

(2) $$\omega_{*}=(1-\pi )\sum_{k=0}^{\infty }\alpha (k)\omega_{*}^{k}=(1-\pi )G(\omega_{*})$$

where *G*(*z*) is the probability generating function of the offspring distribution. We model this as a negative binomial distribution so that $G(z)={\left(1-p/1-pz\right)}^{r}$, but our approach could use other distributions. The solution $\omega_{*}$ to Equation 2 is calculated numerically (supplementary material S1, Supplementary Material online).

The probability that an individual has *d* offspring who are included in the transmission tree is

(3) $$P(d\;\text{offspring}\;\text{included}\;)=\sum_{k=d}^{\infty }\left(\begin{array}{c} k\\ d \end{array}\right)\alpha (k)\omega_{*}^{k-d}{\left(1-\omega_{*}\right)}^{d}$$

In our final expression for P(T|θ), arrived at by induction, each included case will have its own term. For notational simplicity, we define the “modified offspring function” to collect the other parts of this expression:

(4) $$\alpha_{*}(d)=\sum_{k=d}^{\infty }\left(\begin{array}{c} k\\ d \end{array}\right)\alpha (k)\omega_{*}^{k-d}$$

A good approximation is obtained by taking the sum up to a large value. Note that if sampling is complete then *π* = 1 so that $\omega_{*}=0$ and $\alpha_{*}(d)=\alpha (d)$.

We now consider a transmission tree T generated from the model, which is made of *n* nodes corresponding to the included infected individuals, some of whom are sampled and others not. They are indexed by i=1,…,n. Let *s_i_* = 0 if *i* is unsampled and *s_i_* = 1 if *i* is sampled, in which case its sampling time is $t_{i}^{\text{sam}}$. Let $t_{i}^{\text{inf}}$ denote the time when *i* became infected and *d_i_* denote its number of included offspring who are indexed by $j=1\cdots d_{i}$. The probability of T given the parameters *θ* can be obtained by considering the root *ρ* of the tree, which has $d_{\rho }$ offspring, and the subtrees ${\left\{T_{j}\right\}}_{j=1..d_{\rho }}$ corresponding to each offspring. A recursive form of the probability of the transmission tree can then be written as:

(5) $$p_{*}(T|\theta )={\left(1-\pi \right)}^{1-s_{\rho }}{\left(\pi \sigma (t_{\rho }^{\text{sam}}-t_{\rho }^{\text{inf}})\right)}^{s_{\rho }}\sum_{k=d_{\rho }}^{\infty }\left(\left(\begin{array}{c} k\\ d_{\rho } \end{array}\right)\alpha (k)\omega_{*}^{k-d_{\rho }}\prod_{j=1}^{d_{\rho }}\left[p_{*}(T_{j}|\theta )\gamma (t_{j}^{\text{inf}}-t_{\rho }^{\text{inf}})\right]\right).$$

The parameters *θ* appear in the offspring distribution *α*, the generation time density *γ* and the sampling time density *σ*. The terms in the square brackets do not depend on *k*, so that we can rearrange the equation using the modified offspring function $\alpha_{*}$ defined in Equation 4:

(6) $$p_{*}(T|\theta )={\left(1-\pi \right)}^{1-s_{\rho }}{\left(\pi \sigma (t_{\rho }^{\text{sam}}-t_{\rho }^{\text{inf}})\right)}^{s_{\rho }}\alpha_{*}(d_{\rho })\prod_{j=1}^{d_{\rho }}\left[p_{*}(T_{j}|\theta )\gamma (t_{j}^{\text{inf}}-t_{\rho }^{\text{inf}})\right]$$

Finally by induction we obtain the probability of T as a product over all nodes of the transmission tree:

(7) $$p_{*}(T|\theta )=\prod_{i=1}^{n}\left[{\left(1-\pi \right)}^{1-s_{i}}{\left(\pi \sigma (t_{i}^{\text{sam}}-t_{i}^{\text{inf}})\right)}^{s_{i}}\alpha_{*}(d_{i})\prod_{j=1}^{d_{i}}\gamma (t_{j}^{\text{inf}}-t_{i}^{\text{inf}})\right]$$

### Ongoing Outbreak Scenario

We now consider the situation where an outbreak follows the same model as previously described, until some known time *T* where observation stops. Whereas individuals were previously all sampled with the same probability *π*, it is now necessary to account for the fact that individuals who became infected just before *T* have a lower probability of being sampled. More formally, the probability of sampling for an individual infected at time *t* is equal to:

(8) $$\pi_{t}=\pi \int_{0}^{T-t}\sigma (\tau )d\tau$$

Stopping observation at time *T* also affects the probability of being excluded, with all individuals infected at t≥T being excluded since neither they nor any of their descendants can be sampled.

For an individual infected at time *t*, let *ω_t_* be the probability of being excluded. Note that where $t\geq T,\;\omega_{t}=1$. Before that time, *ω_t_* is not constant, but we know that as t→−∞, we should have $\omega_{t}\to \omega_{*}$. We have that:

(9) $$\omega_{t}=(1-\pi_{t})\sum_{k=0}^{\infty }\alpha (k){\left[\int_{0}^{\infty }\gamma (\tau )\omega_{t+\tau }d\tau \right]}^{k}$$

Let ${\bar{\omega }}_{t}=\int_{0}^{\infty }\gamma (\tau )\omega_{t+\tau }d\tau$. Using the generating function *G*(*z*) of the negative binomial distribution of α(k) we have $\omega_{t}=(1-\pi_{t})G({\bar{\omega }}_{t})$. We approximate ${\bar{\omega }}_{t}$ using a numerical integration (supplementary material S1, Supplementary Material online). Good agreement is found with the expected limit $\omega_{-\infty }=\omega_{*}$ where $\omega_{*}$ is given in Equation 2.

As before, we use the modified offspring function to simplify the notation:

(10) $$\alpha_{t}(d)=\sum_{k=d}^{\infty }\left(\begin{array}{c} k\\ d \end{array}\right)\alpha (k){\bar{\omega }}_{t}^{k-d}$$

and obtain a good approximation by taking the sum up to a large value.

With the same recursive reasoning as in the finished outbreak scenario, we have

(11) $$P(T|\theta )=\prod_{i=1}^{n}\left[{\left(1-\pi_{t_{i}^{\text{inf}}}\right)}^{1-s_{i}}{\left(\pi_{t_{i}^{\text{inf}}}\sigma_{t}(t_{i}^{\text{sam}}-t_{i}^{\text{inf}})\right)}^{s_{i}}\alpha_{t_{i}^{\text{inf}}}(d_{i})\prod_{j=1}^{d_{i}}\gamma_{t}(t_{j}^{\text{inf}}-t_{i}^{\text{inf}})\right]$$

where $\sigma_{t}(\tau )$ and $\gamma_{t}(\tau )$ are, respectively, equal to σ(τ) and γ(τ) truncated at time τ=T−t.

### Inference of Transmission Tree Given a Phylogeny

**Fig. 1:**
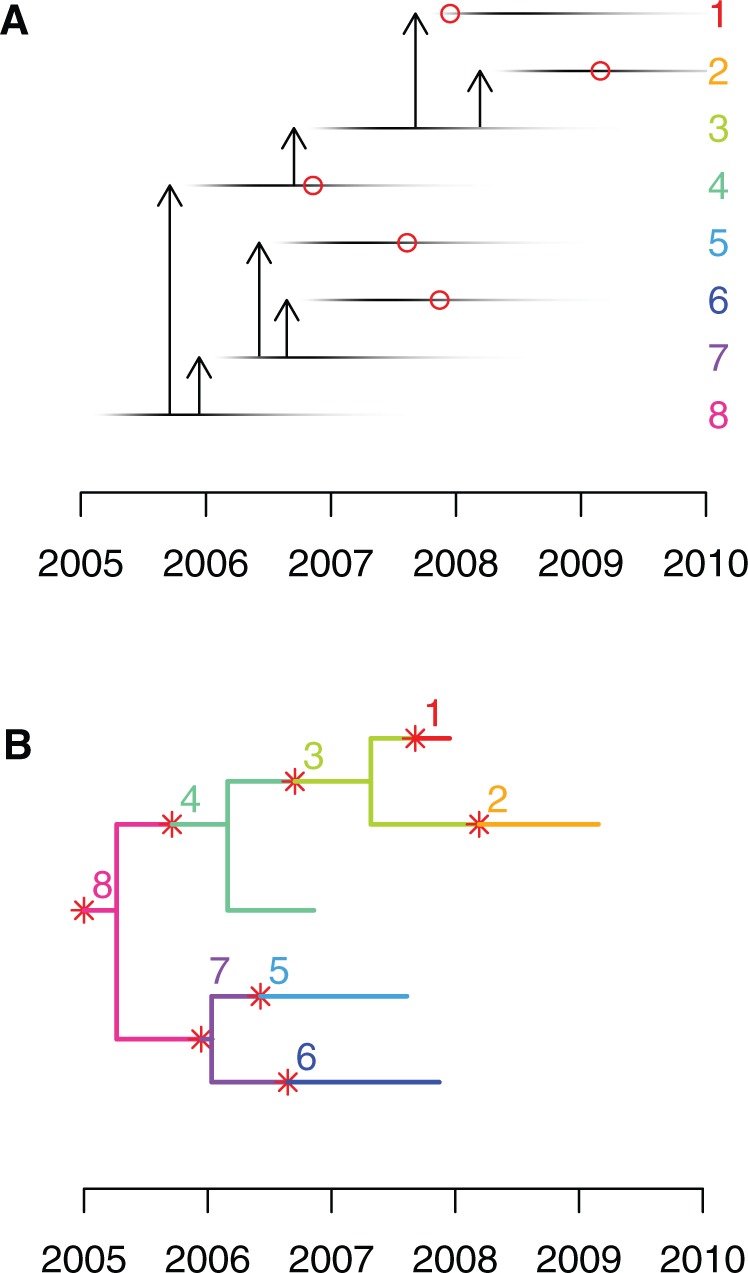
(*A*) An illustrative example of transmission tree, with each horizontal line representing a case, and the darkness of each point representing their changing infectivity over time. Vertical arrows represent transmission from case to case. The red circles indicate which individuals were sampled (1, 2, 4, 5, and 6) and when. (*B*) An example of colored phylogeny which corresponds to the transmission scenario shown in part A. Evolution within each host is shown in a unique color for each individual, as indicated by the labels and on the right-hand side in (*A*). Red stars represent transmission events and correspond to the arrows shown in (*A*). Tips of the phylogeny represent sampled cases as shown by the red circles in (*A*).

The models described above generate transmission trees where each node is an infected individual, each terminal node is a sampled infected individual, and links between nodes represent direct transmission events (fig. 1*A*). Let us now consider that transmission involves the transfer of only a single genomic variant of the pathogen from the donor to recipient (i.e. a complete transmission bottleneck) and that sampling involves sequencing a single genome, randomly selected from the within-host pathogen population. The ancestry of the sequenced genomes can then be described as a phylogeny which is made of several subtrees, each of which corresponds to the evolution within one of the included hosts and describes the ancestral relationship between the genomes transmitted and/or sampled from that host (fig. 1*B*). The probability $P(P|T,N_{e}g)$ of a pathogen phylogeny P given a transmission tree T and within-host effective population size *N_eg_* is therefore equal to the product of the subtree likelihoods for all included hosts (Didelot et al. 2014; Hall et al. 2015), which can be calculated for example under the coalescent model with constant population size *N_eg_* (Kingman 1982; Drummond et al. 2002).

Having defined both P(T|θ) and $P(P|T,N_{e}g)$, we can now perform Bayesian inference of the transmission tree T given a timed phylogeny P using the decomposition in Equation 1. Although a timed phylogeny is not directly available, there are powerful approaches readily available to reconstruct it from genomic data (Drummond et al. 2012; Bouckaert et al. 2014; Biek et al. 2015; To et al. 2016). As in our earlier work (Didelot et al. 2014), we can approach this problem by coloring the phylogeny with one color for each host (fig. 1*B*); however, since we now consider that some hosts may not have been sampled, the number of infected hosts and therefore the number of colors is not known. In other words, the parameter space is not of fixed dimensionality, and exploring it with a Monte–Carlo Markov Chain (MCMC) requires that we include reversible jumps that change the number of hosts in the transmission tree (Green 1995). Our proposal for adding new transmission events is uniformly distributed on the edges of the phylogeny P. Our proposal for removing transmission events is uniformly distributed on the set of transmission events that can be removed without invalidating the transmission tree. In a transmission tree T with *n* hosts and $\sum_{i=1}^{n}s_{i}$ sampled hosts, there are $n-\sum_{i=1}^{n}s_{i}$ such removable transmission events. The Metropolis–Hastings–Green ratio for the MCMC move from T to T′ by adding a transmission event is therefore equal to:

(12) $$\alpha_{T\to T\prime }=\text{min}\left(1,\frac{P(T\prime |\theta )}{P(T|\theta )}\frac{P(P|T\prime ,N_{e}g)}{P(P|T,N_{e}g)}\frac{\left|P\right|}{n+1-\sum_{i=1}^{n}s_{i}}\right)$$

where |P| denotes the sum of the branch lengths of the phylogeny P. Conversely, the acceptance ratio of the MCMC update from T to T′ by removing a transmission event is

(13) $$\alpha_{T\to T\prime }=\text{min}\left(1,\frac{P(T\prime |\theta )}{P(T|\theta )}\frac{P(P|T\prime ,N_{e}g)}{P(P|T,N_{e}g)}\frac{n-\sum_{i=1}^{n}s_{i}}{\left|P\right|}\right).$$

Within each MCMC iteration, additional standard Metropolis–Hastings moves are used to estimate the first parameter *r* of the Negative binomial distribution for the number of offspring [using an Exponential(1) prior], the second parameter *p* of the Negative binomial distribution of the number of offspring (using a Uniform([0,1]) prior), the probability of sampling *π* (using a Uniform([0,1]) prior), and the within-host effective population size *N_eg_* (using an Exponential(1) prior). We implemented our transmission tree inference technique in an R package called TransPhylo. All data and methods used in the study are freely available from https://github.com/xavierdidelot/TransPhylo.

## Results

### Example Application to a Simulated Dataset

**Fig. 2:**
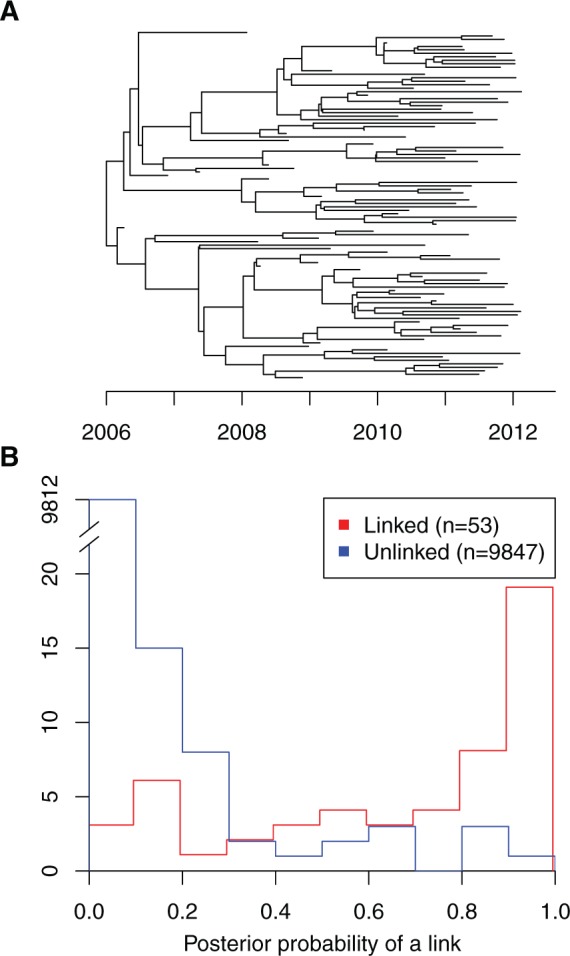
(*A*) Timed phylogeny showing the relationship between 100 genomes sampled with density π=0.5 in a simulated outbreak. (*B*) Distribution of the posterior probability of direct transmission inferred by our algorithm for pairs of individuals in which a link existed in the simulation (red) and pairs of individuals that were not linked (blue).

We simulated an outbreak in which the generation time distribution had a Gamma distribution with a mean of 1 year, and the offspring distribution was a negative binomial with parameters (r=2,p=0.5), such that the reproduction number was *R* = 2. We set the sampling density at π=0.5 with a sampling time distribution identical to the generation time distribution. The simulation was stopped after *n* = 100 genomes had been sampled, which happened at time *T*. The corresponding phylogeny (fig. 2*A*) was used as input of our transmission tree inference algorithm TransPhylo with the date *T* used as described in the “ongoing outbreak scenario” in the Methods section. Performing 10^5^ MCMC iterations took less than an hour on a standard computer. The mean posterior of the sampling proportion *π* was 0.48 with a 95% credibility interval of [0.36, 0.59]. The mean posterior of the reproduction number *R* was 2.168367 with a 95% credibility interval of [1.75, 2.65]. The estimates of these two key parameters of our model are therefore in excellent agreement with the true values used to perform the simulation.

Out of the *n* = 100 sampled individuals, only 53 were infected by another sampled individual; for the majority of these links, our algorithm inferred the existence of the link with high posterior probability, with only nine pairs being given a probability lower than 0.2 and 15 pairs being given a probability lower than 0.5 (fig. 2*B*, red curve). Conversely, for the 9,847 pairs of sampled individuals for which a link did not exist in the simulated data, most were given a very small probability of a link in the posterior distribution of transmission trees, with only nine pairs being given a probability higher than 0.5 (fig. 2*B*, blue curve). If we consider 0.5 as the probability threshold for when transmission was inferred, our method had a specificity (true negative rate) of 99.9% and a sensitivity (true positive rate) of 72%. The area under the receiver-operating characteristic (ROC) curve was 98.97%. These results demonstrate that in this specific example our algorithm was able to infer the correct transmission links with high accuracy, in spite of having information about only a proportion π=0.5 of infected individuals. It should be noted that this application represents a best case scenario, since the phylogeny is known exactly, whereas for real epidemiological investigations it would need to be inferred from sequences, adding noise, and uncertainty.

To test the performance of our algorithm on a smaller dataset, we repeated the same analysis of a simulated dataset with only *n* = 40 sampled individuals. The mean posterior of *π* was 0.53 with credibility interval [0.33, 0.73] and the mean posterior of *R* was 1.95 with 95% credibility interval [1.36, 2.71]. The posterior distributions of these two parameters were therefore centered on the correct values of π=0.5 and *R* = 2, but as expected had larger variance than in the previous example where *n* = 100. Supplementary figure S1, Supplementary Material online shows the posterior probabilities attributed to correct and incorrect transmission links in this simulated dataset.

### Evaluation of Performance Using Multiple Simulated Datasets

**Fig. 3:**
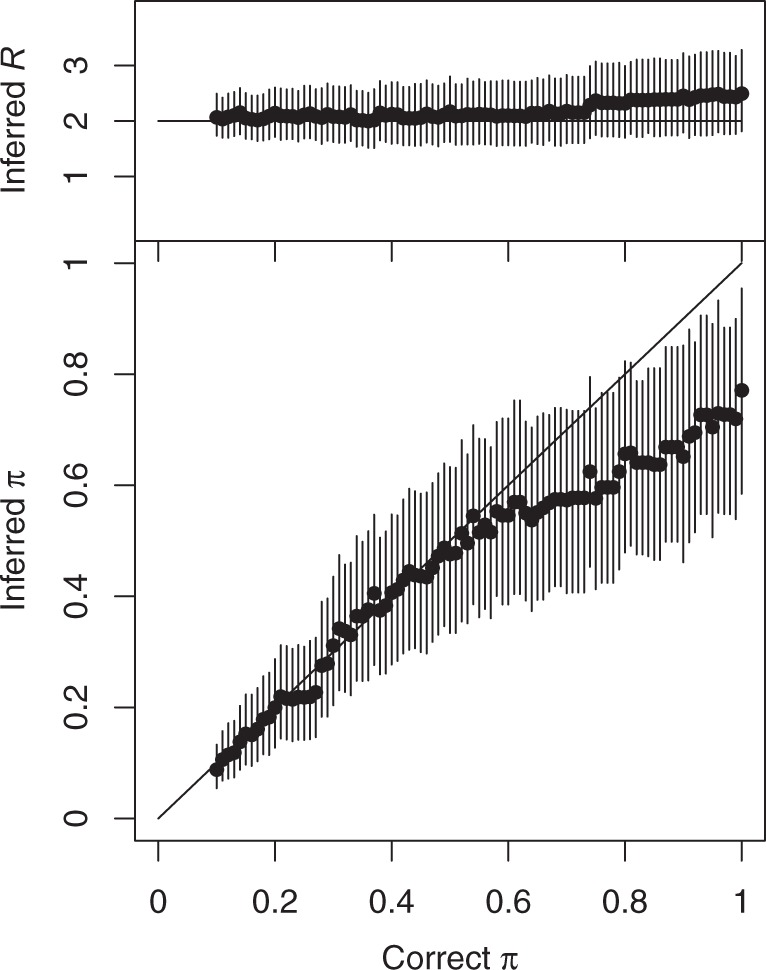
Inferred values of the reproduction number *R* (top) and the sampling proportion *π* (bottom) in simulated datasets for which the correct value of *R* is 2, and the correct value of *π* is increased from 0.1 to 1 (as shown on the *x*-axis). Dots represent the mean of the posterior sample and bars the 95% credibility intervals.

We repeated the simulation described above with *n* = 100 sampled individuals for values of the sampling density *π* varying from 0.1 to 1 by increments of 0.01, whereas leaving the reproduction number constant at *R* = 2. For each of the 90 simulated datasets, we applied our algorithm to estimate the values of both *π* and *R* (fig. 3). We found that the estimate of *R* remained fairly constant as it should, whereas the estimate of *π* increased as the correct value of *π* was increased. There was no sign of a bias in the estimates up to π=0.6, but higher values of *π* were consistently underestimated, with the value of *R* being slightly overestimated in compensation. We attribute this bias to the difficulty in assessing with certainty whether all cases have been sampled in a transmission chain, since there always remains a possibility that an unsampled individual may have acted as intermediate. In other words, the data are not very informative about *π* when *π* is high, so that datasets simulated for example with π=0.9 and π=0.8 look highly similar whereas datasets with π=0.1 and π=0.2 look different. This small bias also reflects our choice of prior for *π*, which was uniform between 0 and 1, and the fact that only 100 genomes were used in each simulation.

**Fig. 4:**
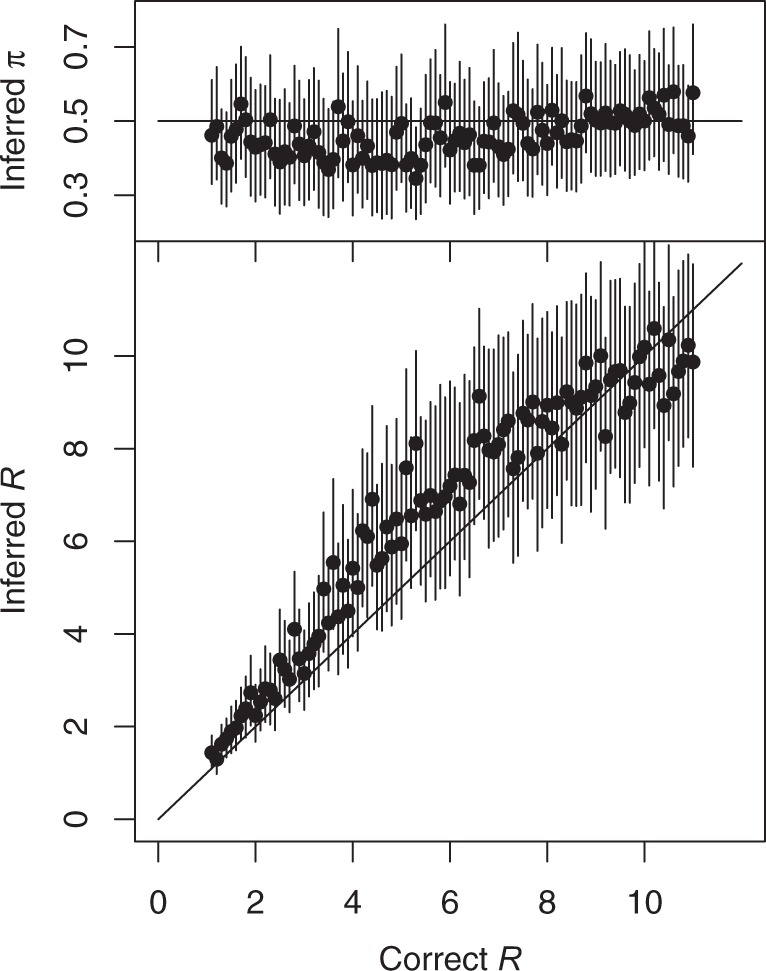
Inferred values of the sampling proportion *π* (top) and the reproduction number *R* (bottom) in simulated datasets for which the correct value of *π* is 0.5, and the correct value of *R* is increased from 1 to 11 (as shown on the *x*-axis). Dots represent the mean of the posterior sample and bars the 95% credibility intervals.

We also performed simulations in the converse situation where the sampling density was kept constant at π=0.5 but the reproduction number was increased from *R* = 1 to *R* = 11 by increments of 0.1. For each of the 100 simulated datasets, our method was applied and the inferred values of *π* and *R* were recorded (fig. 4). Although there was once again a slight bias toward underestimating the sampling density *π*, its 95% credibility intervals always covered the correct value of π=0.5. The inferred values of *R* were accordingly overall slightly upward biased, although they followed almost linearly the correct values used for simulation. The 95% credibility intervals for *R* almost always included the correct values. We conclude from these results that our algorithm performs well despite being tested in difficult situations, with only 100 sampled genomes, unknown proportions of unsampled cases, uninformative priors, and simulations using very large intervals of values of the sampling density *π* and reproduction number *R*. A small outbreak with high-sampling density and a larger outbreak with lower sampling density can often look similar, especially in the first stages of an ongoing outbreak, but our algorithm is able to distinguish between these two scenarios with good accuracy.

### Application to a *Mycobacteriumtuberculosis* Outbreak Dataset

We applied the method to a previously reported tuberculosis outbreak (Roetzer et al. 2013). We used BEAST (Drummond et al. 2012) to infer a timed phylogeny from the published data, using a coalescent model with constant population size and a strict molecular clock model. BEAST was run for 10^7^ iterations, with the parameter state recorded every 1,000 iterations and the first 10% discarded as burn-in. A maximum clade credibility tree was built to summarize the posterior sample of trees (supplementary fig. S2, Supplementary Material online). This phylogenetic tree was then used as input of our algorithm TransPhylo to investigate transmission (cf. supplementary fig. S3, Supplementary Material online for a comparison of results when using a sample of posterior phylogenies). In determining the best priors for the densities of the times between becoming infected and infecting others (the generation time) and between becoming infected and becoming known to the health care system (sampling time), we considered both clinical aspects of tuberculosis disease and aspects of the epidemiological investigation. The outbreak lasted 13 years, during which active case finding was used to identify individuals with prior exposure to known cases. An early report on this outbreak (Diel et al. 2004) noted that many cases were identified for reasons not connected to their tuberculosis infection, such as presenting to health care with other symptoms, to obtain a health certificate, or to enter a detox program. We therefore used a Gamma distribution for the sampling time density, with a shape parameter 1.1 and rate 0.4. The generation time for tuberculosis should reflect a chance of relatively rapid progression from infection to active disease and hence to the opportunity to infect others, but also a possibility of infection leading to a long latent period before progression (Barry et al. 2009). We therefore used a Gamma distribution with shape parameter 1.3 and rate parameter 0.3 for the generation time density. We ran four separate chains with 10^5^ iterations each. We computed both the Gelman–Rubin (GR) diagnostic and effective sample size (ESS) for three parameters: The basic reproduction number *R*, the sampling probability *π* and the within-host effective population size *N_eg_*. We found that GR was always below 1.05 and ESS always above 100, indicative of good convergence and mixing properties of the MCMC (Kass et al. 1998). Only the first of the four separate MCMC runs was used for further analysis; its MCMC traces are shown in supplementary figure S4, Supplementary Material online and the posterior predictive distribution of the number of observed cases is shown in supplementary figure S5, Supplementary Material online.

**Fig. 5:**
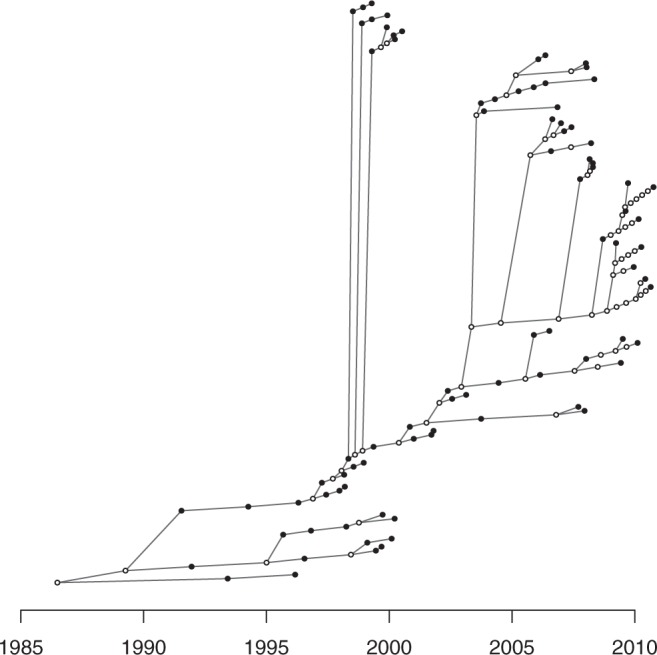
Consensus transmission tree for the tuberculosis outbreak. To avoid confusion between this transmission tree and a phylogenetic tree, the layout is different from the way phylogenetic trees are usually represented. Dots represent individuals with on the *x*-axis the posterior mean time of infection. The *y*-axis is arbitrary. Filled dots represent sampled individuals and unfilled dots represent unsampled inferred individuals.

**Fig. 6:**
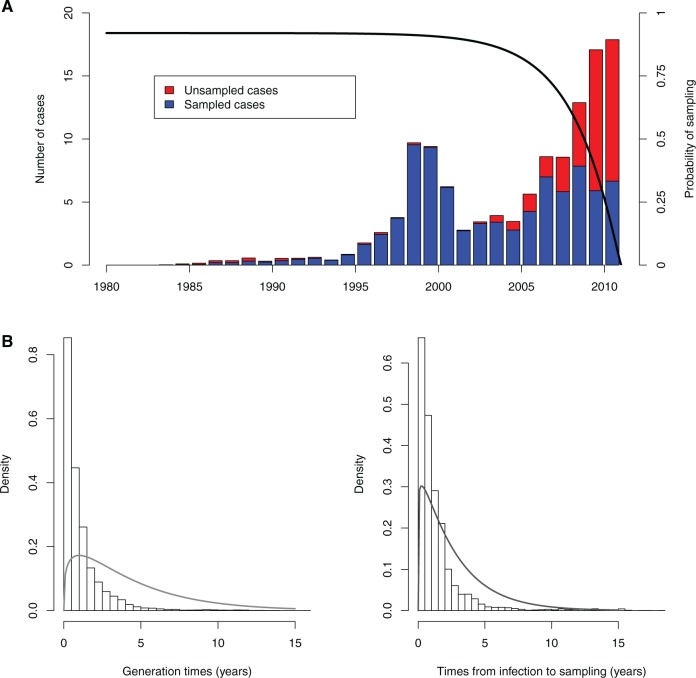
(*A*) Outbreak plot showing the numbers of sampled and unsampled cases through time in the posterior sample of transmission trees. Although the posterior estimate of *π* is 0.93, predicting that cases would eventually be detected with high probability, in the time period just before sampling ended, the inferred transmission trees contain a number of unsampled cases. The solid line represents the probability of sampling cases as a function of their infection time, given that observation stops at *T* = 2011. (*B*) Posterior generation times and times between infection and sampling. Bars show histograms of the posterior quantities and solid lines show the related prior densities.

Figure 5 shows the consensus transmission tree for this real-world tuberculosis outbreak (Roetzer et al. 2013), and figure 6*A* shows the inferred numbers of unsampled cases along with the reported cases through time. The date of infection of the index case was estimated to be in June 1986, with 95% credibility interval from June 1983 to December 1987. Although most cases were sampled, reflecting a robust public health investigation, we estimate that early in the outbreak, several unsampled individuals were contributing to transmission (simulations varying *R* with fixed *π* suggest that our method is not biased toward inference of more early unsampled cases, though their numbers are variable, cf. supplementary fig. S6, Supplementary Material online). During this period, the two major clades of the phylogeny diverged. Figure 6*A* recapitulates the two major waves of the outbreak—an early peak around 1998 and a second pulse from 2005 onwards—each with a small portion of inferred unsampled cases. Although the number of unsampled individuals was small, the method does allocate key transmission events to unsampled cases, particularly early in the outbreak, suggesting that screening and investigation earlier in the outbreak was not as comprehensive as it eventually became. This is to be expected, as outbreak management efforts typically intensify as the number of cases grows.

Figure 6*B* shows the posterior times between an individual becoming infected and infecting others (the generation time) and the posterior time intervals between infection and sampling (the infectious period), with priors shown in grey. Our observed generation times are variable, which reflects the clinical history of tuberculosis—an infection that can progress rapidly to active, infectious disease or that can have an asymptomatic, noninfectious latent period of variable length. We used a Gamma distribution as a prior, with mode strictly greater than zero, but the posterior generation times have a mode closer to zero, suggesting a relatively high portion of those who go on to infect others have a rapid progression to from infection to active disease. It is important to note that the posterior generation times are only an indicator of the inferred natural history of tuberculosis *among those with active disease who were sampled*; individuals who were infected but did not progress to active disease and those who never presented to care and were not sampled do not appear in the dataset, and those who did not infect others do not appear in the cases behind the inferred generation times. The mean posterior generation time was 1.0 years with a standard deviation of 1.36 years. The posterior times between becoming infected and becoming known to health authorities also differ from the prior assumption; they have a mean of 1.4 years and standard deviation of 2 years. Sampling times are distinct from the prior but are affected by a change in the prior assumption.

Where inferred infectors are sampled cases with associated clinical and/or epidemiological data, an advantage of our approach is that it allows comparison of the relative contributions of different groups of individuals to the burden of transmission. Supplementary figure S7, Supplementary Material online shows the inferred per-case transmission stratified by several characteristics of the cases (Roetzer et al. 2013): Individuals’ AFB smear status (a measure of how many bacilli are found in their sputum, if any), HIV status, abuse of alcohol or other drugs, and whether the individual had a permanent domestic residence. Our method did not detect significant differences in secondary infections arising from smear-positive and -negative cases, between substance users and nonsubstance users, and between stably or transiently housed individuals. However, consistent with the hypothesis that HIV-positive patients tend to be less infectious with tuberculosis (Huang et al. 2014), we find that HIV-positive individuals transmitted somewhat fewer cases on average than HIV-negative individuals. Owing to the small number of HIV-positive cases—only five individuals were HIV-positive in this data—the estimates are much more variable than the estimates for HIV-negative cases. Many more clinical or demographic factors might impact transmissions, such as the presence of cavitary disease and the reported number of social contacts, but these data were unavailable for the present analysis.

Results in supplementary figure S7, Supplementary Material online do not reflect differences in transmission rates given contact with others, because we do not know about exposures that did not result in infection. We also do not have information about behaviors that might modulate transmission. For example, if smear-positive cases sought and obtained treatment more rapidly than smear-negative cases, or were more unwell and had more limited activities, their transmission rate per contact could be higher than their smear-negative counterparts but they might still contribute fewer onward transmissions. The posterior sampling density is π=0.93 with a standard deviation of 0.05, consistent with a very densely sampled outbreak in a high-resourced setting with good case finding.

## Discussion

We have described a new methodology for reconstructing who infected whom based on genomic data from an infectious disease outbreak. The novelty of this approach, which extends our earlier work in the area, is that it now accounts for both the possibility of some cases not having been sampled and the possibility that more cases may occur in the future. Addressing these issues overcomes key hurdles in using genomic data to reconstruct disease transmission events during a real-time public health response. In these situations, a case may not be sequenced due to a lack of clinical specimen or otherwise sequenceable material, whereas cases might go unsampled for various reasons, including subclinical, or asymptomatic, infections for which an individual may not seek care, or a diagnosis in another jurisdiction. Furthermore, following early proof-of-concept retrospective studies, genomic epidemiology is now being used to prospectively understand outbreaks, as in the recent outbreak of Ebola (Gire et al. 2014). Allowing inference before the end of the outbreak turns our method into a real-time, actionable approach.

Our methodology is based on an explicit transmission model that makes a number of assumptions, some of which could be relaxed if required by specific applications. A first example is the fact that we consider a branching process with reproduction number *R* remaining constant throughout the outbreak. This contrasts with our previous work (Didelot et al. 2014) assuming an SIR model, in which *R* decreases over time due to depletion of susceptible hosts. The new branching process has many advantages over the previous SIR model, allowing more flexibility in the distributions of offspring number, sampling time and generation time, as well as having useful mathematical properties which we used to derive the probability of a transmission tree. However, there are situations where the reproduction number varies over time and quantifying these variations is of great epidemiological importance (Cori et al. 2013). Application of our methodology as in stands in such a situation could still be insightful as temporal trends in the posterior distribution for the number of offspring can be significant even if our prior model is constant over time. Temporal variation could be explicitly incorporated in the prior model relatively easily, for instance by assuming stepwise changes or some predetermined parametric function for *R*(*t*), the parameters of which could be jointly estimated in our Bayesian framework. A second example concerns the observation of cases, which we assumed to happen with probability π(t) for an individual infected at time *t*, with π(t) reflecting both the impossibility of observing cases happening after the time *T* when observation stops, and the lower probability of observing cases soon before *T* (Equation 8). It is often difficult in epidemiological studies to know the real function π(t), but in situations where, for example, surveillance did not start before a certain date, the function π(t) we used here could be updated to reflect this. Other assumptions in our model would be more difficult to relax, such as the complete transmission bottleneck which considers that only a single pathogen variant is transmitted from the donor to the recipient of each transmission event.

A key feature of our methodology is that it proceeds in two steps—first, genomic data are used to reconstruct a phylogenetic tree, and second, likely transmission events given the phylogeny are inferred. There are both advantages and disadvantages to this approach, compared with the more theoretically accurate joint inference of phylogenetic and transmission trees (Hall et al. 2015; De Maio et al. 2016; Klinkenberg et al. 2016). Our two-step approach makes it difficult to pass the uncertainty in the phylogenetic reconstruction on to the transmission analysis. This is especially relevant if the time-labeled phylogeny is inferred not using a point estimation procedure (Fourment and Holmes 2014; To et al. 2016), but rather with a Bayesian sampling method (Drummond et al. 2012; Bouckaert et al. 2014). In this case, applying the transmission analysis separately to a sample of trees from the phylogenetic posterior can help account for uncertainty (Didelot et al. 2014). However, two problems remain: How to choose the tree prior in the phylogenetic reconstruction and how to combine the results from the separate transmission analyses. A solution may be to consider that the phylogenetic trees sampled in the first step are coming from a biased distribution, which can be corrected for using importance sampling in the second step, such that the separate transmission analyses are correctly aggregated and the prior used in the first step is nullified (Meligkotsidou and Fearnhead 2007). It should also be noted that our two-step approach has significant advantages both computationally and conceptually. Computationally, we were able to analyze outbreaks with hundreds of cases in a matter of hours. Conceptually, working with a fixed phylogeny allows us to explore much more complex models for transmission trees, such as the partially sampled and ongoing scenarios. To date, no other transmission inference approach can handle these difficult scenarios. Another advantage of our two-step approach is that it allows relatively easy detection of separate introductions of a pathogen in the population of interest (Jombart et al. 2014; Worby et al. 2016). Under such a scenario, there would be several clusters in the phylogenetic tree each of which corresponds to a separate entry followed by local transmission. The expected genetic distance between separate introductions varies depending on the pathogen, size of the population under study, and global epidemiological properties, but clusters corresponding to clearly separate introductions are usually easy to infer by simple observation of the phylogenetic tree (Nelson et al. 2006; Didelot et al. 2012; Holt et al. 2012; He et al. 2013; Didelot et al. 2015). These genetic clusters can then be analyzed independently to reconstruct local transmission events.

We have previously applied earlier versions of our approach to understanding a complex tuberculosis outbreak in a largely homeless Canadian population (Didelot et al. 2014; Hatherell et al. 2016), showing how it reveals key individuals contributing to transmission and how its ability to time infection events can be used to declare a waning tuberculosis outbreak truly over. Here, we demonstrate our new methodology’s ability to identify unsampled cases. Finding such cases is critically important for tuberculosis control—not only does it allow us to seek out these individuals and connect them with treatment, but also it allows us to extend our case-funding efforts to include a larger proportion of potentially exposed individuals. In our present analysis of the Hamburg dataset, we found that the generation time was relatively rapid, with the majority of infected individuals progressing to active disease and infecting others doing so within 2 years, with many progressing to active disease almost immediately. This is important data for outbreak management—if borne out by further reconstructions, it suggests a bound for the time over which an individual who has been exposed to tuberculosis should be followed up.

Although the ability to more confidently infer both the direction and the timing of a disease transmission event represents a powerful tool for understanding an outbreak’s dynamics, it raises critical ethical and legal issues. The HIV community has struggled with the use of phylogenetics in the criminal prosecution of HIV transmission for several years—most hold that phylogenetics can exclude the possibility of transmission but not prove that transmission occurred (Romero-Severson et al. 2016). The introduction of approaches such as the one presented here complicate the landscape by introducing the possibility of proof via high posterior probabilities of individual transmission events. Were this technique to be used in criminal prosecution, extensive model-based and real-world validation would be required, and a set of rigorous guidelines would have to be established, including the appropriate use of controls and standards related to genome sequencing and informatic processing (Budowle et al. 2014). We speculate that for rapidly evolving pathogens such as the RNA viruses, inference methods that report the direction of transmission may eventually appear on the judicial stage. For more clonal bacterial pathogens such as tuberculosis, validation is likely to reveal that the degree of uncertainty is too high to definitively rule in transmission events, and that the technique is best suited for excluding the possibility of person-to-person transmission. Beyond the judicial domain, one must also consider whether the academic publication of transmission networks is being carried out in a manner that protects patient privacy. In settings with a low burden of a specific disease, the affiliations of the authors, the name of the disease, and simple metadata such as sampling time might be sufficient for knowledgeable individuals to identify cases within the network. Given the growing research into transmission inference, guidance around appropriate metadata release and anonymization is clearly warranted.

In conclusion, we present a new method TransPhylo for the automated inference of person-to-person disease transmission events from pathogen genomic data, one which accounts for the complex and variable nature of sampling cases during an outbreak. When coupled to the routine genomic surveillance of key pathogens now in place at many public health agencies, such as Public Health England’s new genomic approach to tuberculosis diagnosis and laboratory characterization (Pankhurst et al. 2016), our method has the potential to rapidly suggest the contact network underlying an outbreak. Given the significant resources associated with a contact investigation, any tool that can quickly assist in prioritizing individuals for follow-up is an important contribution to the public health domain.

## Supplementary Material

Supplementary data are available at *Molecular Biology and Evolution* online.

## Supplementary Material

Supplementary Data
