# Supplementary material for: Genomic Infectious Disease Epidemiology in Partially Sampled and Ongoing Outbreaks
Source: Mol Biol Evol. 2017 Jan 19;34(4):997–1007. doi: 10.1093/molbev/msw275 (PMC5850352; doi:10.1093/molbev/msw275)
Supplement: Supplementary Data [file msw275_supp.pdf]

Supplementary material for “Genomic infectious disease epidemiology in partially sampled and ongoing outbreaks”

Xavier Didelot[1], Christophe Fraser[1,2], Jennifer Gardy[3,4], Caroline Colijn[5]

1 Department of Infectious Disease Epidemiology, Imperial College London, Norfolk Place, London, W2 1PG, United Kingdom

2 Oxford Big Data Institute, Li Ka Shing Centre for Health Information and Discovery, Nuffield Department of Medicine, University of Oxford, Oxford OX3 7BN, United Kingdom

3 Communicable Disease Prevention and Control Services, British Columbia Centre for Disease Control, Vancouver, British Columbia, Canada

4 School of Population and Public Health, University of British Columbia, Vancouver, British Columbia, Canada

5 Department of Mathematics, Imperial College, London SW7 2AZ, UK

Numerical calculation of ω_*

In the finished outbreak scenario, the probability of being excluded, which means being unsampled and having all descendants unsampled, is given in Equation 2. We calculate ω_* numerically by solving Equation 2 using the R command uniroot over the interval [0,1].

Numerical approach to ω_t

In the ongoing outbreak scenario, let ω_t be the probability of being excluded, which means being unsampled and having all descendants unsampled, conditional on being infected at time t. As $t \to -\infty$, $\omega_t \to \omega_*$ which is the solution to Equation 2 described above. However, as the study ends at a finite time T, we know that $\omega_T = 1$ because a case infected at T will be excluded.

We do not know the times of an individual's descendants, but we still condition on the total number of descendants. Integrating those out, we have

$$\omega_t = (1 - \pi_t) \sum_{k=0}^{\infty} \alpha(k) \prod_{j=1}^{k} \left[\int_t^{\infty} \gamma(\tau_j - t) \omega_{\tau_j} d\tau_j \right] \tag{S1}$$

Let the term in square brackets be $\bar{\omega}_t$. We need to determine what this function is because ultimately, we need it to compute the probability of having k included descendants.
We have (with G the probability generating function of the negative binomial offspring distribution, and p and r its parameters)

$$\omega_t = (1 - \pi_t) G(\bar{\omega}_t) = (1 - \pi_t) \left(\frac{1-p}{1 - p\bar{\omega}_t} \right)^r . \tag{S2}$$

We have $\omega_t = 1$ for $t \geq T$, so

$$\bar{\omega}_t = \int_t^{\infty} \gamma(\tau - t)\omega(\tau) d\tau = \int_t^{T} \gamma(\tau - t)\omega(\tau) d\tau + \int_T^{\infty} \gamma(\tau - t) d\tau$$

We substitute this into Equation S2. We use the trapezoid method for the first term, and the second term we can compute explicitly: $\int_{T-t}^{\infty} \gamma(u) du \equiv F(t)$. Let $t_i = T - i\Delta t$. The trapezoid method gives:

$$\int_t^T \gamma(\tau - t)\omega(\tau) d\tau \approx \sum_{i=0}^{k} c_i \gamma((k-i)\Delta t)\omega(t_i)\Delta t$$

where $c_i = 1$ unless $i = 0$ or $i = k$, where $c_i = 1/2$. The k'th term drops out because $\gamma(0) = 0$ by assumption, so:

$$\omega(t_k) \approx (1 - \pi_t) \left(\frac{1-p}{1 - pF(t) - p\sum_{i=0}^{k-1} c_i \gamma((k-i)\Delta t)\omega(t_i)\Delta t} \right)^r \tag{S3}$$

This is straightforward to compute with iteration. We find that $\omega_t \to \omega_*$ as $t \to -\infty$. This gives the probability of being excluded (ie being unsampled and having no sampled descendants), conditional on having been infected at time t.

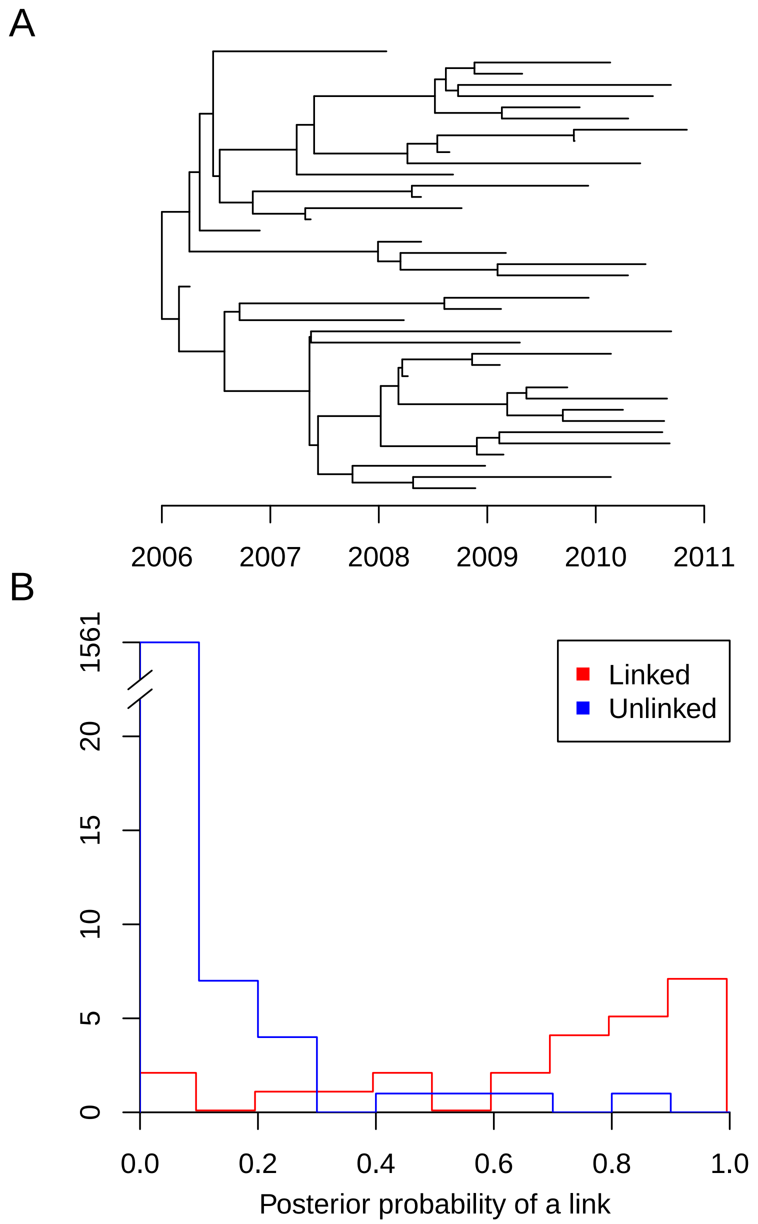

Figure S1. Equivalent to Figure 2 when analysing a simulated dataset with only $n = 40$ sampled individuals, $\pi = 0.5$ and $R = 2$.

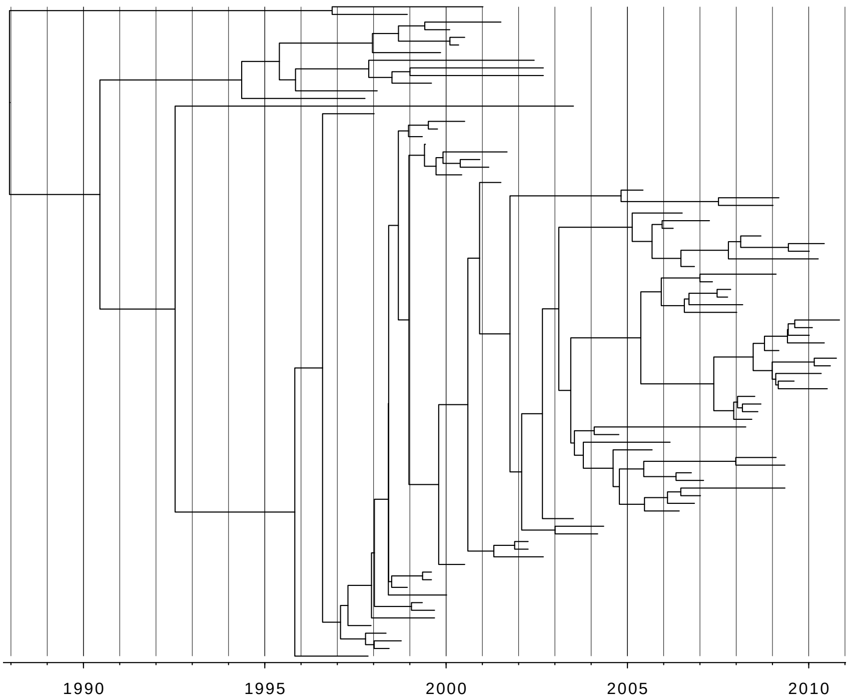

Figure S2. Phylogenetic tree used in the tuberculosis application. This tree was computed using maximum clade credibility (MCC) on the posterior sample of trees returned by BEAST.

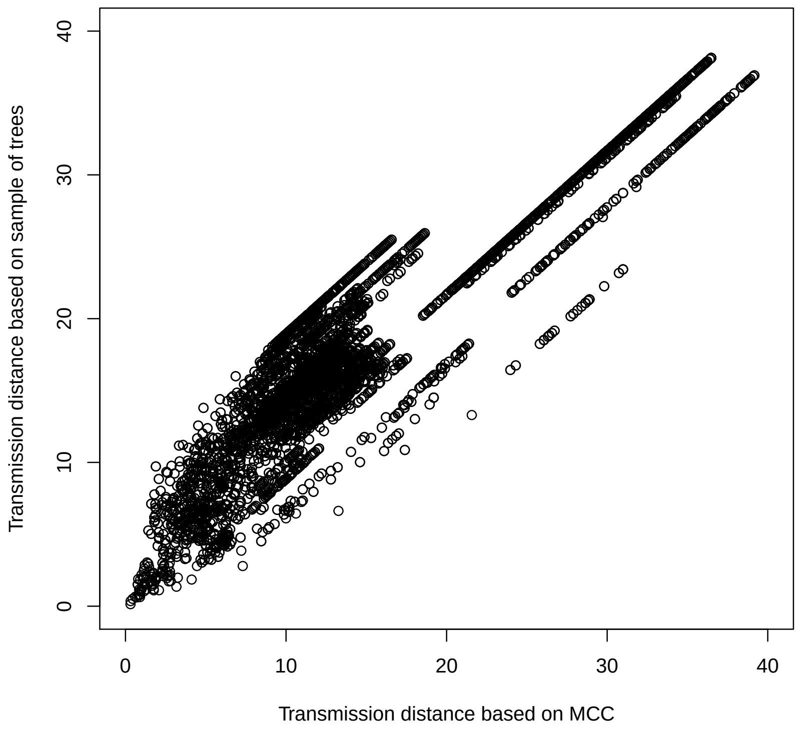

Figure S3. Comparison of transmission results based on the maximum clade credibility (MCC) tree and a sample of 100 posterior trees. For a given transmission tree, the "transmission distance" between two sampled individuals is defined as the sum of the two sampling dates minus twice the date of infection of most recent common infector. This distance is shown for all pairs of sampled individuals in the output based on the MCC tree (x-axis) and averaged in outputs based on a sample of 100 posterior trees (y-axis).

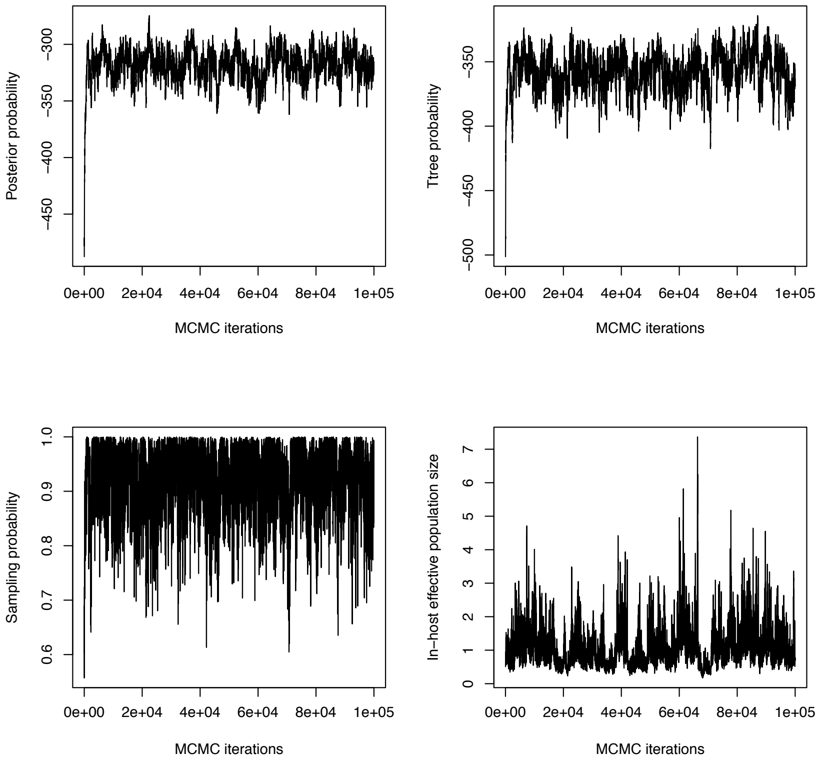

Figure S4. MCMC traces in the tuberculosis application.

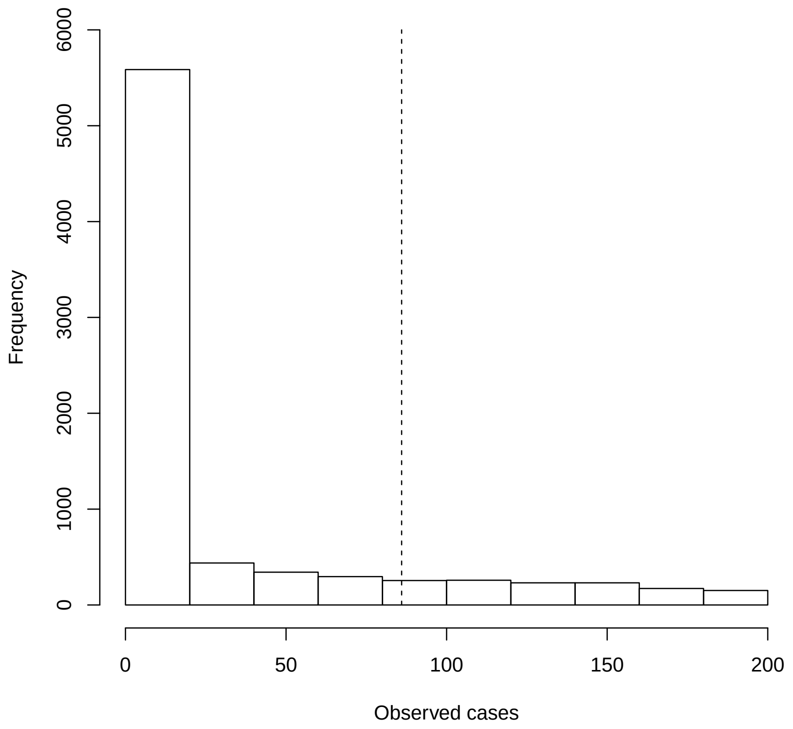

Figure S5. Posterior predictive distribution for the number of observed cases in the tuberculosis application, with the dotted line representing the actual number of observed cases.

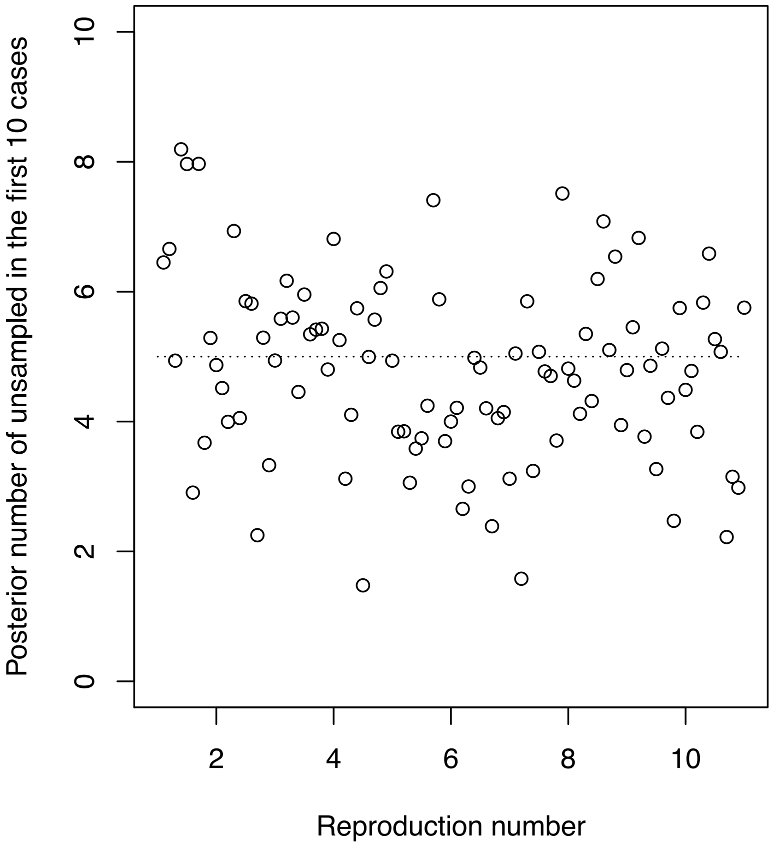

Figure S6. Test of bias in inference of early unsampled cases. This figure is based on the set of 100 simulations used for Figure 4 where the sampling probability is $\pi = 0.5$ and the reproduction number R is varied between 1 and 11 (x-axis). For each of these simulations, we calculated the inferred number of unsampled individuals amongst the first 10 cases of the outbreak (y-axis).

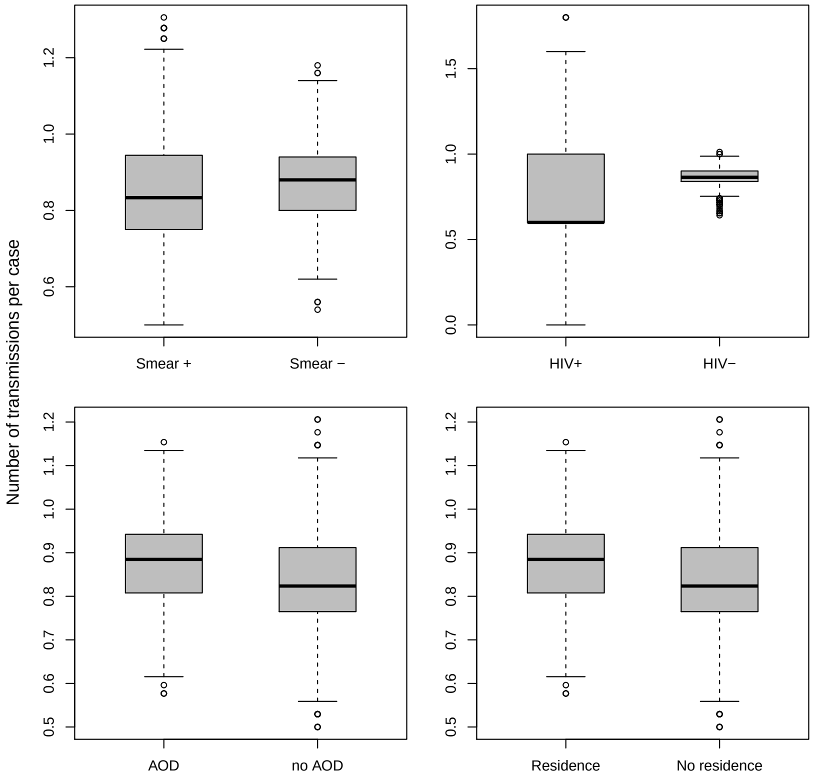

Figure S7. How direct transmissions among sampled cases are affected by potential risk factors. Among the inferred transmission events between sampled individuals, we computed the number of transmissions by (eg) smear-positive and smear-negative individuals divided by the number of smear-positive and -negative individuals to obtain a per-individual average number of transmissions. We computed this for 1000 samples from the posterior MCMC chain (chosen uniformly at random from the latter half of the posterior).
